# Kineispathy

**Published:** 1852-01

**Authors:** 


ARTICLE VII.

### KINEISPATHY.

A new system of medical practice has been introduced into Europe, and it may naturally be expected that it will be imported, and sooner or later practised among us. It would not be strange were it to supercede and take the place of homœopathy, to which it is assimilated in other points besides a common lack of science or reason. It certainly is superior on the score of economy, for though the doses to be shaken in the former are infinitesimal and therefore portable and cheap, in the latter no doses at all are required, and all the mysterious movements and "shakings" are to be accomplished on the sick body itself! The originator of this improved system appears to have been a Swedish fencing master by the name of Ling, who is represented, in the Edinburgh Monthly Journal, to have been an universal genius. He was successively a graduate in theology, a volunteer in the Danish navy, (in spite of gout in his arm,) a lecturer on old Norse poetry, history and mythology, a professor of fencing and gymnastics, a student of anatomy, physiology and other sciences, a writer of poetry, and, withal, "a man of high moral tone, pious, sincere and and honest," and died in 1839 with the honors of knighthood upon him. His qualifications are therefore unquestionable! All that Ling himself appears to have really accomplished, and probably all that he claimed at first, was set forth in a work published by him, and may be considered as merely an improvement in the practice of gymnastics and calisthenics. Upon this has been engrafted the system of quackery alluded to above. M. Roth, M. D., of London, who comes before us clothed with Ling's mantle, has sent out an octavo of 300 pages, devoted to the treatment of disease by "movements," alias Kineispathy. His interpretation of the term is as follows:

"By the word *movement*, in a medical and hygienic sense, is to be understood every change of position and difference of form, determined by time and amount, in the whole body, or in any part of it, and which may be produced by the organism itself, or by any animate or inanimate mechanical agent."

In accordance with this definition, there are a great variety of movements; quite as many as there are dilutions and potencies in the homœopathic system; and each and all possess great power over the human body, as is rendered plain by another quotation:

"Whatever exists in our body, either as a part of it or as a foreign substance, must at a certain moment have a definite shape; therefore every change of the space in one part necessarily produces a corresponding one in the surrounding tissues; a change is hence propagated to the most remote parts of the body, and which depends, with respect to its form, upon the amount of the alteration produced by the first movement."

Lest any one should still be in the dark, however, respecting what kinesipathy really is, we copy the full definition of one of the movements and its effects. It is called the

"*Chopping Movement.*---Chopping consists in alternate short blows, produced by the external side of both the operator's hands. Choppings are principally used on the posterior surface of the trunk, chest, and also on the limbs. If it is desirable that the succession produced by this movement shall be less and softer, then the chopping is done with the external edges of the two little fingers, while the other fingers are spread apart, but not spasmodically fast, so that they act also by striking upon the little finger.

"Chopping may be confined to one only, or may be exercised on a larger surface, by constantly moving the position of the hands. The chopping is called a *longitudinal* one, if the hands are moved in the longitudinal direction of the trunk or of the limb; and a *transversal* one if the blows are executed across the limbs.

"*Effect.*—Choppings produce generally a venous absorption in the capillary texture, not only of the external skin and the tendinous expansions, but also, if more strongly used, in the muscles and bones; in imperfectly paralyzed muscles they excite the innervention both of the motory and sensitive fibres. If directed on the lower extremities, on the soles, they act very well in hæmorrhoidal complaints, headache, &c. On the chest or along the spine, they are efficacious specific movements in certain complaints of the chest, partly by their direct influence on the muscles of the chest, partly by the tremulous, passive vibration communicated to the lungs."

Then there is the "shaking movement," the "rising-up movement," she letting-down movement," "transversal chopping," "vibration," &c., &c., which we have not room to describe. These "movements" are all claimed as a remedy in acute as well as chronic diseases. In gonnorrhœa, even, cases are brought forward to show their great efficacy. Can quackery and imposture "further go?" It does really seem as though we might hope that "things will come right at last," when such a multitude of absurdities and inconsistencies are countenanced and supported by those who break sway from, or who never have entered, the ranks of legitimate and scientific practice.—*Boston Med. Jour.*

